# Improving sexual health for HIV patients by providing a combination of integrated public health and hospital care services; a one-group pre- and post test intervention comparison

**DOI:** 10.1186/1471-2458-12-1118

**Published:** 2012-12-27

**Authors:** Nicole HTM Dukers-Muijrers, Carlijn Somers, Christian JPA Hoebe, Selwyn H Lowe, Anne-Marie EJWM Niekamp, Astrid Oude Lashof, Cathrien AMVH Bruggeman, Hubertus JM Vrijhoef

**Affiliations:** 1 Department of Sexual Health, Infectious Diseases, and Environmental Health, South Limburg Public Health Service, P.O. Box 2022, 6160 HA, Geleen, The Netherlands; 2 Department of Medical Microbiology, School of Public Health and Primary Care (CAPHRI), Maastricht University Medical Centre (MUMC+), PO Box 5800, 6202 AZ, Maastricht, The Netherlands; 3 Department of Integrated Care, Maastricht University Medical Centre, PO Box 5800, 6202 AZ, Maastricht, The Netherlands; 4 Department of Internal Medicine, section Infectious Diseases, Maastricht University Medical Centre (MUMC+), PO Box 5800, 6202 AZ, Maastricht, The Netherlands; 5 School of Social and Behavioural Sciences, Tilburg University, PO Box 90153, 5000 LE, Tilburg, the Netherlands

**Keywords:** HIV, Quality of care, Services integration, Public health care, Hospital care, STI

## Abstract

**Background:**

Hospital HIV care and public sexual health care (a Sexual Health Care Centre) services were integrated to provide sexual health counselling and sexually transmitted infections (STIs) testing and treatment (sexual health care) to larger numbers of HIV patients. Services, need and usage were assessed using a patient perspective, which is a key factor for the success of service integration.

**Methods:**

The study design was a one-group pre-test and post-test comparison of 447 HIV-infected heterosexual individuals and men who have sex with men (MSM) attending a hospital-based HIV centre serving the southern region of the Netherlands. The intervention offered comprehensive sexual health care using an integrated care approach. The main outcomes were intervention uptake, patients’ pre-test care needs (n=254), and quality rating.

**Results:**

Pre intervention, 43% of the patients wanted to discuss sexual health (51% MSM; 30% heterosexuals). Of these patients, 12% to 35% reported regular coverage, and up to 25% never discussed sexual health topics at their HIV care visits. Of the patients, 24% used our intervention. Usage was higher among patients who previously expressed a need to discuss sexual health. Most patients who used the integrated services were new users of public health services. STIs were detected in 13% of MSM and in none of the heterosexuals. The quality of care was rated good.

**Conclusions:**

The HIV patients in our study generally considered sexual health important, but the regular counselling and testing at the HIV care visit was insufficient. The integration of public health and hospital services benefited both care sectors and their patients by addressing sexual health questions, detecting STIs, and conducting partner notification. Successful sexual health care uptake requires increased awareness among patients about their care options as well as a cultural shift among care providers.

## Background

The number of HIV patients receiving HIV care is growing worldwide. Due to the availability of effective treatments, we are now facing an AIDS-free generation of HIV-infected men and women. As stated by the WHO, these patients’ rights to maintain their sexual health must be respected, protected and fulfilled [1]. These rights include the right to achieve ‘the highest attainable standard of sexual health, including access to sexual and reproductive health care services’, the right to ‘seek, receive and impart information related to sexuality’ and the right to ‘pursue a satisfying, safe and pleasurable sexual life’. HIV-infected persons are considered to have unmet needs with respect to sexual health care, i.e. counselling about safe sex, relationships, reproductive health and pregnancy, and testing and treatment for sexually transmitted infections (STIs) [2-6]. The current care systems fail to effectively address the sexual health of HIV patients, primarily because of the highly fragmented organisation of these care systems [7]. Historically, most countries in Europe have organised HIV care and sexual health care into separate systems (i.e., hospital and public health systems). HIV care is largely hospital based and focused on HIV treatment, and there is growing awareness of the need for improved sexual health care in HIV care settings. An example is the adoption of routine STI testing guidelines in the HIV care setting in the US, the UK and the Netherlands. However, compliance with guidelines in the HIV care setting is inadequate [8-11]. In some cases, specific recommendations regarding testing at multiple anatomic sites or repeated testing are lacking. These testing approaches are essential STI control strategies that are often included in standard operating procedures at public health STI clinics [12]. STIs, including *Neisseria gonorrhoeae* (NG) and *Chlamydia trachomatis* (CT), increase the risk of HIV transmission [13-16]. It is important to note that sexual health care is not restricted to the provision of STI testing. Sexual health care also entails extensive counselling, partner notification, and treatment, services that are a part of the regular care offered at public health sexual health care centres [12].

Brickley et al. reviewed 10 intervention studies with a pre-post or multi-arm design that examined the addition of sexual health care to existing HIV services [17-26]. These studies as well as a recent study on the addition of nurse-led self-screening for STIs [6]. have demonstrated positive effects on condom use, contraceptive use, STI screening, and quality of services. Only three of these studies evaluated a comprehensive sexual health care package that addressed both women and men [24-26]. None of these studies examined the pre-test needs of patients, a key factor in the successful integration of services. Important gaps remain in the research regarding the best approaches for addressing the needs and choices of HIV patients [17]. Church and Lewin [7] proposed that policy development and provider training, while important, will not necessarily lead to the practical integration of care. They state that for optimal integration, care must shift from task orientation and functional separation to patient-centred approaches. This shift requires organisational and cultural change focusing on the patient’s perspective. Therefore, to improve the patient orientation of services, scientific research should be integrated into the care-focused clinical setting by collecting and using epidemiological and qualitative findings to optimise practice.

This study aimed to close the gap in sexual health care by implementing and evaluating a policy change regarding the combination of public health care and hospital care in an innovative, integrated STI /HIV care structure serving male and female HIV patients in the Netherlands. Using a health impact assessment framework [27], the potential health effects of this intervention were evaluated based on the patient’s perspective. Practical recommendations are provided for stakeholders, such as providers of care to HIV patients in the public and hospital care sectors.

## Methods

A health impact assessment framework [27] was followed that included (1) screening, (2) scoping, (3) evaluating impact, and (4) providing recommendations.

### Screening

To determine whether a health impact assessment was needed and how such an assessment would best be undertaken, several multidisciplinary team meetings were held with stakeholders involved in policy, practice and research (HIV care doctors and nurse practitioners, public health care doctors and nurses, researchers, and managers). This process resulted a list of current practical barriers for sexual health care provision to HIV patients and included a lack of HIV patient attendance to public health care settings (sexual health care centres), in spite of their comprehensive sexual health care services. In practice, sexual health care in the HIV care hospital setting appeared difficult due to limited STI testing guidelines, financial barriers, the lack of a public health perspective and priority given to public health goals, limited provider time and a lack of specialised STI testing and sexual health counselling expertise. It also appeared that there was no systematic data collection to understand patients’ needs. Current sexual health care provision in the hospital care setting largely depends on the personal initiatives of HIV care providers. HIV patients do not receive optimal sexual health care at the majority of Dutch HIV centres. Insufficient STI prevention and care for HIV patients results in new STIs and serious individual complications and creates an enormous potential for transmission, thereby increasing the population’s STI/HIV burden.

### Scope: integrating policy change into public health and hospital care (intervention)

A consensus was reached in the multidisciplinary team to establish a policy change regarding collaboration between public health and hospital care. This collaboration was considered likely to have a positive impact on the HIV-positive population. The intervention entailed the integration of sexual health care services (sexual health care counselling and STI testing) into routine HIV care services. This intervention was chosen because it was expected to be able to overcome current practical barriers (such as time, financial issues and expertise) with relatively minimal effort. With this ongoing collaboration (synergy I model by Lasker) [28], we aimed to improve health care by coordinating sexual health and HIV care services for HIV-infected individuals.

The intervention was tested at the HIV Centre from the Maastricht University Medical Centre between November 2009 and November 2010. The HIV Centre serves a region of approximately 0.6 million inhabitants and provided HIV care to 447 adult patients in active follow-up during the study period. All patients were eligible for the intervention and were included when they visited the hospital for their usual consultations with their HIV care providers (nurse practitioner and medical doctor). The HIV Centre took an active role by motivating and referring patients to experienced public health care nurses for sexual health care visits, which involved a 10-minute counselling session tailored to the HIV patient and testing for CT and NG at urogenital, anorectal and oropharyngeal body locations. The public health care nurses were employed by the regional Sexual Health Care Centre and could be consulted directly after the HIV care visit in the hospital (a nurse was available in the adjacent room on some week days) or at the Sexual Health Care Centre outpatient location located near the hospital. These nurses had specifically been trained to address sensitive sexual health issues in HIV-positive people (using motivational interviewing techniques), to provide multiple anatomic site STI testing, and partner notification. The public health nurses provided extensive sexual health care with a focus on the public health scope according to national Sexual Health Care Centre guidelines [12].

In addition to implementing practical measures and ensuring geographic proximity, a long preparation phase was used to develop commitment and a shared policy between the institutes. Before and during the intervention, regular team meetings were held to ensure that goals and protocols continued to match.

**Figure 1:**
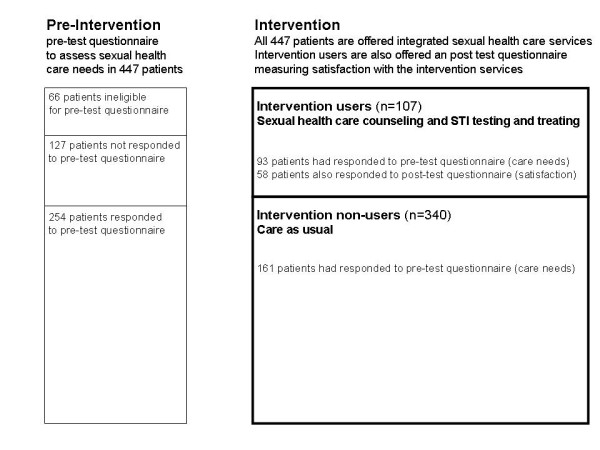
Description of the study; response to the questionnaires and intervention uptake among 447 HIV patients receiving HIV care who were offered integrated sexual health care services.

Questionnaires to assess patients’ needs (pre), care satisfaction (post) and STI diagnosis data (post) were used to evaluate the impact of the intervention. Figure 1 shows the responses to the questionnaires for the study population.

#### Pre-test patient questionnaire on sexual health care needs

A self-administered questionnaire, blinded to the HIV care providers, was administered before patients were offered integrated services. The questionnaire included an extended version of the validated QUOTE instrument that was composed of 17 items on HIV care and 3 items on STI care. These items were rated for importance on a 4-point Likert scale [29]. Further, several questions were included on the perceived need for sexual health care and coverage at the HIV care visit. Patients were ineligible to complete this questionnaire if they did not understand the Dutch language, had psychiatric contraindications, or were considered too ill. Of the 381 eligible patients, 254 (67%) returned the questionnaire. Respondents (median age, 47 years) were older (p<0.01) than non-respondents (44 years).

#### Post-test questionnaire on satisfaction with new sexual health care services

The patients who used integrated services provided by a public health care nurse were asked to complete a self-administered questionnaire on quality of the care providers using a 4-point Likert scale. Fifty-eight (54%) of the intervention users completed the questionnaire. The respondent group was more likely to include MSM than the non-respondent group (p=0.03). The respondents were similar to non-respondents with respect to age, test location (hospital or Sexual Health Care Centre), STI prevalence and pre-test care importance scores.

### Evaluating impact

The study design was a one-group pre-test post-test comparison based on the use of the intervention. The impact of the intervention was evaluated for the following outcomes: (1) patients’ sexual health care needs, (2) uptake of the intervention, (3) the yield in terms of STIs identified, and (4) patient satisfaction and the quality of improvement for the new services.

For outcome (1), the mean importance scores and the proportion of patients expressing a need for sexual health care were assessed and compared between HIV transmission groups using a one-way ANOVA, chi-square test statistics, and McNemar’s test (when comparing topic coverage between health care providers). For outcome (2), logistic regression analysis was applied to assess determinants for service usage. The evaluated determinants included transmission group, age and needs data obtained from the pre-test questionnaire. For outcome (3), chi-square test statistics were used to compare the STI prevalence between the transmission groups. For outcome (4), individual performance scores (from the post-test questionnaire) were subtracted from importance scores (from the pre-test questionnaire) to indicate the quality of improvement (below zero: low quality of improvement) and to identify care items to be improved (servqual method). Because the numbers did not allow meaningful comparisons between transmission groups, such comparisons were not performed. We considered a p value <0.05 statistically significant. Analyses were performed with SPSS 17.0 (IBM Corporation, Somers, New York, USA). Ethical approval was obtained from the Medical Ethical Committee of the Maastricht University Medical Centre (09-4-011; 09-4-011.6).

### Recommendations

Following the results of the intervention evaluation and the multidisciplinary team meetings, practical recommendations were formulated.

## Results

### Pre-test sexual health care needs assessment for HIV patients

#### Importance of HIV and STI care items

The respondents were categorised hierarchically into four transmission groups: (1) intravenous drug users (IDUs) and non-IDUs, which included (2) MSM, (3) women and (4) heterosexual men. In each group, items related to information, privacy and expertise were rated as the most important HIV care-related items. On average, the sexual health care items were rated as important (Table 1). The mean scores for the HIV- and STI-related items overall were higher for MSM than for IDUs, women and heterosexual men (p<0.01).

**Table 1:** Pre-intervention mean importance scores of HIV and STI care-related items (scale: 1 not important, 2 somewhat important, 3 important, 4 very important) for 254 HIV-infected patients receiving hospital-based HIV care

	**Total (n=254)**	**MSM (n=162)**	**IDUs (n=26)**	**Women (n=33)**	**Heterosexual Men (n=33)**
All 17 HIV care items	3.2	3.3	3.2	3.1	3.0
Most important items					
Information on medication	3.6	3.7	3.5	3.6	3.4
Expertise on HIV	3.6	3.7	3.6	3.6	3.4
Privacy regarding HIV	3.6	3.7	3.3	3.3	3.5
Understandable language	3.5	3.5	3.4	3.5	3.4
All 3 STI-related items	3.2	3.4	3.1	2.9	3.1
Privacy regarding STIs	3.5	3.6	3.4	3.2	3.5
Expertise on STIs	3.2	3.3	3.0	3.1	3.1
Opportunity for STI screening	3.0	3.1	2.9	2.6	2.7

#### Expressed need for sexual health care

Forty-three per cent (n=110) of participants had sexual health questions that they wanted to discuss with a health care provider. This proportion was higher for MSM (51%, n=83) than for IDUs (23% (n=6) p<0.01), women (33% (n=11) p=0.05) and heterosexual men (30% (n=10) p=0.03). The topics mentioned most frequently included information on STIs and related symptoms (Table 2). Notably, the majority of women with questions mentioned safe sex and sexual relationships as topics.

**Table 2:** Reported topics for 110 HIV patients who wanted to discuss sexual health with a care provider prior to service integration

	**Total (n=110)**	**MSM (n=83)**	**IDUs (n=6)**	**Women (n=11)**	**Heterosexual Men (n=10)**
	% (n)	% (n)	% (n)	% (n)	% (n)
STI-related symptoms	55 (60)	61 (51)	33 (2)	27 (3)	40 (4)
Information on STIs	51 (56)	51 (42)	67 (4)	55 (6)	40 (4)
Sexual problems	44 (48)	45 (37)	17 (1)	46 (5)	50 (5)
Safe sex information	41 (45)	35 (29)	50 (3)	91 (10)	30 (3)
Relationship and sex	34 (37)	30 (25)	33 (2)	73 (8)	20 (2)
Negative sexual experiences	18 (20)	18 (15)	0 (0)	36 (4)	10 (1)
Contraception	16 (18)	12 (10)	33 (2)	46 (5)	10 (1)
Pregnancy	8 (9)	2 (2)	17 (1)	27 (3)	30 (3)

#### Experienced coverage of topics during HIV care visits

Among the patients who wanted to discuss a specific topic, up to 25% had not discussed this topic during their HIV care visits (Table 3). Over two-thirds of patients with questions had not regularly discussed the corresponding topics with any of their HIV care providers. These topics were discussed more regularly with HIV nurse practitioners than with HIV care medical doctors, but the difference was statistically significant only for STI-related symptoms (p=0.02). The coverage of sexual health care topics at the HIV care visits was higher for the 110 patients who wanted to discuss their sexual health than for those patients who did not want to discuss their sexual health. In the former group, more patients regularly discussed STIs, and fewer reported never having discussed STIs, sexual problems, symptoms, contraception, pregnancy or negative experiences (all p<0.05) (data not shown).

**Table 3:** Discussion of sexual health care topics during the HIV care consultation by HIV nurse practitioners and HIV care medical doctors for 110 HIV patients receiving HIV care who indicated that they wanted to discuss the topic

	**Discussed with an HIV nurse practitioner**			**Discussed with an HIV care medical doctor**			**Discussed at the HIV care visit**		
	**Never**	**Sometimes**	**Regularly**	**Never**	**Sometimes**	**Regularly**	**Never**	**Sometimes**	**Regularly**
Topics that patients wanted to discuss	% (n)	% (n)	% (n)	% (n)	% (n)	% (n)	% (n)	% (n)	% (n)
STI-related symptoms	20 (12)	52 (30)	28 (16)	25 (15)	61 (36)	14 (8)	18 (11)	55 (33)	27 (16)
Information on STIs	24 (13)	48 (26)	28 (15)	23 (12)	57 (30)	21 (11)	13 (7)	52 (28)	35 (19)
Sexual problems	17 (8)	58 (28)	25 (12)	37 (17)	50 (23)	13 (6)	13 (6)	57 (27)	30 (13)
Safe sex information	14 (6)	60 (25)	26 (11)	20 (8)	59 (24)	22 (9)	14 (6)	52 (22)	34 (14)
Relationship and sex	11 (4)	67 (24)	22 (8)	23 (8)	60 (21)	17 (6)	11 (4)	61 (22)	28 (10)
Negative sexual experiences	25 (5)	60 (12)	15 (3)	58 (11)	37 (7)	5 (1)	25 (5)	60 (12)	15 (3)
Contraception	18 (3)	47 (8)	35 (6)	13 (2)	69 (11)	19 (3)	6 (1)	59 (10)	35 (6)
Pregnancy	11 (1)	71 (5)	14 (1)	0	100 (8)	0	0	88 (7)	12 (1)

#### Rating of providers regarding delivered sexual health care

The mean quality grade (0–10) for received sexual health care was 8 for both HIV nurse practitioners and HIV care medical doctors. When patients who wanted to discuss their sexual health were asked which type of provider they preferred to discuss such topics with, pre, 58% (n=61) stated their HIV nurse practitioner, 22% (n=23) stated their HIV care medical doctor, and the remainder stated other care providers or had no preference. Pre, 65% (n=73) of patients preferred to receive sexual health care in combination with their HIV care visit.

### Integration of public health and hospital services: intervention impact

#### Use of the intervention and determinants

Of all 447 patients receiving HIV care, 107 (24%) used the new services approach; 95 (87%) of them had not previously visited the Sexual Health Care Centre. The remainder of the HIV patients received sexual health care from their HIV care provider as usual. Of the patients who used the integrated services, 81% (n=86) were MSM, 10% (n=11) were heterosexual men, 9% (n=10) were women, and none were IDUs.

Determinants associated with intervention usage were identified based on the pre-test questionnaire data from 93 intervention users and 161 intervention non-users for whom data were available. Intervention users were more likely to be MSM and were more likely to have questions about sexual health (55% (n=51) vs. 37% (n=59)), particularly questions about STI-related symptoms (36% (n=33) vs. 17% (n=27)), than patients who did not use the intervention. Users rated the mean importance of STI care items higher (mean score 3.4 vs. 3.2) than non-users (all p<0.05). Usage was not significantly associated with age, the preferred type of care provider, the preferred care location, the need to discuss topics other than STI symptoms, or topic coverage at the HIV care visit among patients with sexual health questions.

#### STI diagnosis among those who used the intervention

Among MSM, the prevalences of CT and NG were 13% (n=11) and 2% (n=2), respectively. None of these patients were diagnosed with syphilis. The majority (69%; n=9) of STI positive patients had their STIs diagnosed at anorectal or oropharyngeal sites. All but one case were asymptomatic, and no STIs were found in the heterosexual groups.

#### Post-test satisfaction and quality improvement among those who used the intervention

According to the 58 service users with available post-test data, both the public health care provider and HIV care providers scored sufficiently well on all evaluated items relevant to sexual health care services (Table 4). The main self-reported reasons for usage included certainty (48% (n=28)), convenience (43% (n=25)), and risk behaviour (28% (n=16)). Forty-four patients (76%) intended to use the public health services again. Four patients did not want to use the services from the Sexual Health Care Centre in the future because they felt that they were at low risk or preferred STI screening by their HIV care medical doctor. Ten patients did not provide information on their intended future usage.

**Table 4:** Importance of topics and quality of improvement scores for care providers regarding HIV and sexual health care items as reported by 58 HIV patients who used integrated services, i.e., comprehensive sexual health care provided by public health nurses

		**Quality of improvement**		
	**Mean importance**	**Public health nurse**	**HIV nurse practitioner**	**HIV care medical doctor**
Privacy regarding STI outcomes	3.6	−0.2	−0.3	−0.3
Information in understandable language	3.6	−0.1	−0.2	−0.3
Expertise on STIs	3.4	−0.3	−0.2	−0.3
Opportunity for STI screening	3.3	−0.5	−0.2	−0.3
Take me seriously	3.3	−0.2	−0.3	−0.3
Cooperate with other care providers	3.3	−0.2	−0.5	−0.5
Sufficient time for consultation	3.2	−0.6	−0.6	−0.7
Openness to conversation on sexual health	3.0	−0.7	−0.4	−0.7

Importance was rated on a 4-point scale: 1 not important, 2 somewhat important, 3 important, 4 very important.

The quality of improvement was calculated by subtracting provider performance (4-point scale; 1=never to 4=always) from the patient importance score; quality of improvement scores below zero represent no need for improvement.

## Discussion

To improve sexual health care for HIV patients, patients were provided with easy access to high-quality public health sexual health care (counselling and comprehensive STI testing) during their HIV care visits. An evaluation of the impact of this policy change showed that (1) patients express a need for sexual health care; (2) one-quarter of HIV patients used the integrated sexual health care services; (3) a substantial number of asymptomatic, mostly anorectal and oropharyngeal, STIs were diagnosed in MSM; and (4) patients were satisfied with the care offered.

### Sexual health care needs

Our needs assessment, which preceded service integration, revealed that approximately half of MSM and one-third of heterosexual HIV patients wanted to discuss their sexual health. This finding confirms the importance of sexual health counselling as part of a comprehensive sexual health care package for HIV patients. Our findings highlight some specific care components for different HIV sub-populations. For example, patients in our study wanted to discuss STI-related topics, and safe sex and relationships were mentioned frequently, especially by women. Our study confirms that the counselling practices in the HIV care setting are suboptimal [2-4,7]; up to one-quarter of patients who wanted to discuss a specific topic did not discuss the issue, and only one-third regularly discussed sexual health. It should be noted that the current study did not assess the needs of critically ill patients or those who did not understand the Dutch language. These patients’ needs for sexual health care may be different.

### Use of integrated sexual health care services

We subsequently offered patients comprehensive sexual health care through an integrated hospital and public health services approach. To our knowledge, only three comparable studies on service integration -two including males- have been reported, and all demonstrated improved quality of the services provided to HIV patients [24-26]. Patients used integrated services because of convenience, certainty, and risky sexual behaviour. These patients were more likely to be MSM, and they wanted to talk about their sexual health. The further identification of those who would benefit most from extra services (synergy III model by Lasker) is needed to allow more cost-effective triage of patients to public health services [28].

The reasons that patients did not use extra services are not known but may include both provider- and patient-related reasons. For example, it is likely that some patients were more motivated than other patients to attend integrated services [6]. HIV care providers sometimes felt reluctant to refer patients (‘did not want to impose’), and the implicit perception of low need may have contributed [8]. Another important drawback was the absence of sustained leadership, which acts as a consistent motivator in the HIV care setting to keep the integrated services option on the busy clinical agenda [28]. Patients were referred in the first several days after an inter-sectoral research meeting, but referrals subsequently waned. It may be that patients simply did not have the time to attend, as some mentioned. Most patients were new to public health services and may need to gain experience with the care options to become aware of their own needs and the benefits of other care options.

On a systems level, the integration of care services led to a substantial increase in the number of HIV-positive patients who received sexual health care. The number of patients served by public health care services increased from less than 20 to over 100.

### Intervention components: sexual health care counselling and STIs diagnosed

All patients received extensive counselling and testing. The benefits of STI testing appeared to be greatest for HIV-infected MSM because the prevalence of asymptomatic STIs was substantial in this group. This finding is in agreement with the results of other studies of MSM HIV patient populations, which showed a prevalence of asymptomatic STIs of approximately 17% [6,30]. These cases would most likely have been missed during regular HIV hospital care visits, for which the current guidelines only advise yearly screening for syphilis and screening ‘on indication’ when a patient reports symptoms [8,31]. In the regular HIV care setting, screening is predominantly urogenital. However, the majority of STIs detected in HIV-infected MSM in our study and in other studies were found at anorectal and oropharyngeal sites [6,32]. The current study does not provide insight into the determinants of the higher STI prevalence in MSM. Such determinants may include a higher prevalence of partnership concurrency and more age-disassortative mixing than is common for heterosexuals [33]. No STI cases were found in our tested population of heterosexuals, and only 1.5%-2.1% of STIs were reported among other heterosexual HIV patients in the Netherlands and the UK [26,32]. Standard STI screening may prove to not be an efficient strategy for heterosexuals. Nevertheless, providing both heterosexuals and MSM with comprehensive sexual health care services is necessary given that a substantial part of all patients had questions on sexual health, and some reported additional sexual problems when attending integrated services.

### Satisfaction

Patients rated the quality of received care as sufficient, and the majority of patients indicated that they would use these sexual health care services again.

### Barriers and facilitators for service collaboration

Our chosen approach of collaboration between clinical patient care and public health care facilitates multidisciplinary work and contributes to sustainable partnerships. It can be defined as a synergy I model based on Lasker’s models of medicine and public health collaboration [28]. This collaboration is ongoing and aims to improve health care by coordinating STI, sexual health, and HIV care services for individuals. In addition to the practical changes that were implemented, a long preparation phase allowed for the development of commitment and a shared policy between the institutes. In the current project, several established factors were shown to be major facilitators of service collaboration [28]: the geographic proximity of the services, shared protocols and joint agendas, quality assurance, the sharing of professional information, and trust and respect of the partners. However, even in a case in which compelling need and willingness were expressed and dialogue was maintained between two health sectors with a longstanding history of collaboration, there were many challenges in maintaining collaboration and keeping the services integrated at all levels. The failure to solve difficulties in cross-sectoral research limited the evaluation of our study results to some extent. Nevertheless, the response to the pre-test questionnaire was substantial (67%), and important insights were achieved regarding specific patient needs. It will be a challenge for providers to join their expertise and at the same time time maintain their specific (HIV care and sexual health care) competences in patient care.

### Recommendations

* To achieve and sustain collaboration between hospital and public health services, periodic multidisciplinary team meetings are needed to maintain sexual health care as a topic on the joint agenda, to create opportunities for improvement, and to achieve a cultural shift in care provision.

* Comprehensive sexual health care should include sexual health care counselling tailored to the specific needs of HIV patients and should include standard STI screening at urogenital, anorectal and oropharyngeal sites for MSM.

* The active engagement of HIV care providers is strongly needed to achieve greater coverage of sexual care topics during HIV care visits, to motivate patients to attend additional sexual health care services, and to develop a level of trust with new care providers (such as public health care providers).

* The collection and analysis of scientific data is essential to deliver better care. We recommend periodic assessment of quality of life and needs related to sexual health in HIV care settings. Identifying and addressing the barriers that patients and providers face in obtaining and delivering care will be essential for tailoring and improving the provision of services.

## Conclusions

HIV patients express a need for comprehensive sexual health care, but they are underserved in this respect. The coordination of individual-level hospital and public health services is challenging but feasible. This coordination will allow essential determinants of health to be addressed that go beyond clinical HIV care, such as personal risk behaviours and needs, the management of sexual health problems, and the use of health services. Successful sexual health care uptake among HIV patients requires increased awareness among patients regarding their care options and their needs as well as a cultural shift in hospital and public health organisations to act outside of their own specialisations.

## Competing interest

The authors declare that they have no competing interests.

## Authors’ contributions

ND designed the study, performed the statistical analysis and wrote the manuscript. CS contributed to the acquisition of the data, performed statistical analyses and helped draft the manuscript. BV participated in the design of the study and helped draft the manuscript. All authors participated in the design of the study and the interpretation of the results and have read and approved the final manuscript.

This contents of paper were presented in part at NCHIV, Amsterdam, the Netherlands, on October 28, 2010 and at ESCAIDE, Lisbon, Portugal, on November 11–13, 2010.

## Pre-publication history

The pre-publication history for this paper can be accessed here:

http://www.biomedcentral.com/1471-2458/12/1118/prepub
